# Supplementary figures and images for: Integrated Multi-Omics Analysis Reveals Immune and Metabolic Dysregulation in a Restraint Stress-Induced Depression Model
Source: Biomedicines. 2025 Sep 6;13(9):2183. doi: 10.3390/biomedicines13092183 (PMC12467129; doi:10.3390/biomedicines13092183)

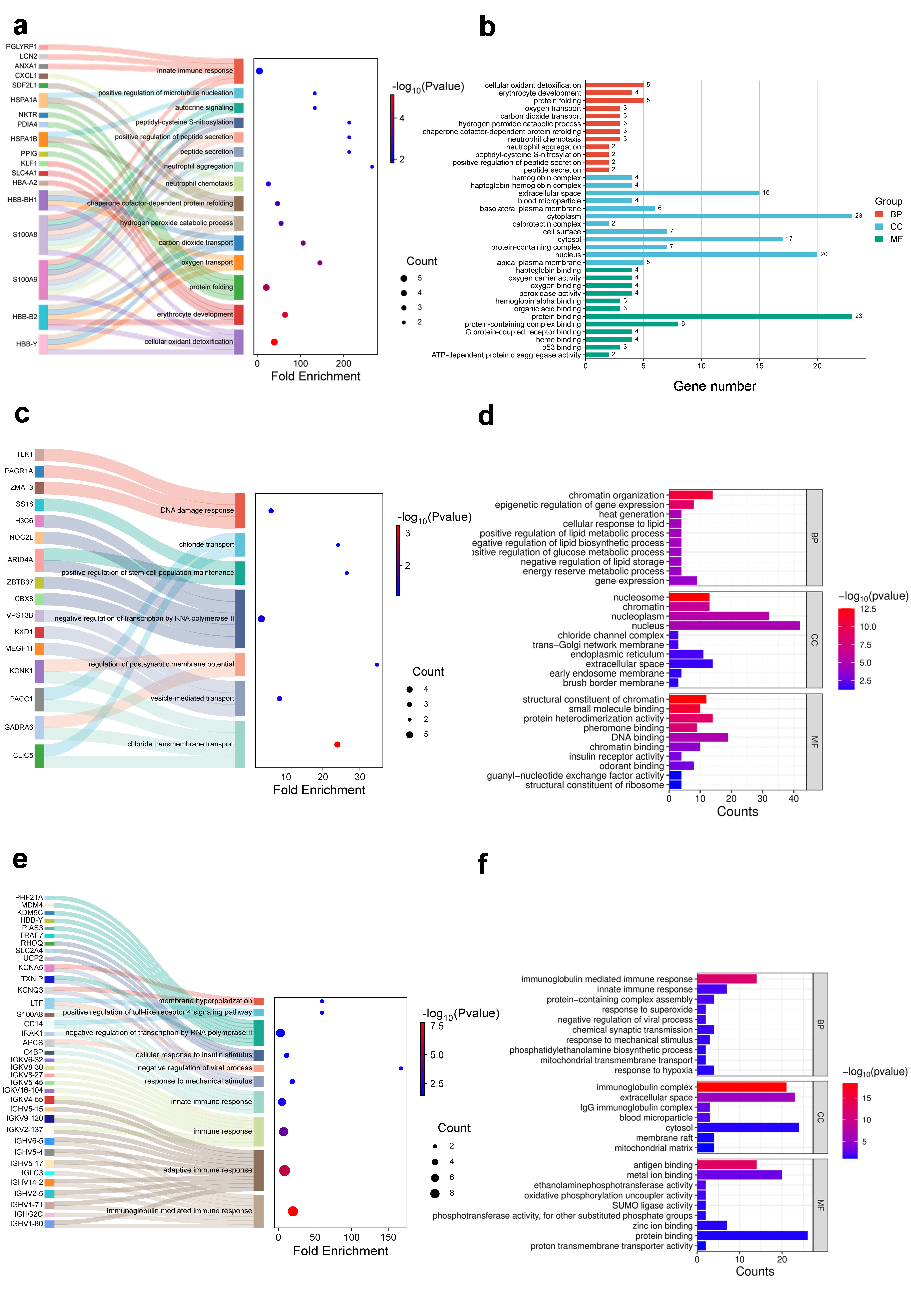

Supplement: Supplementary file 1 [file biomedicines-13-02183-s001.zip › Fig. S1.tif]

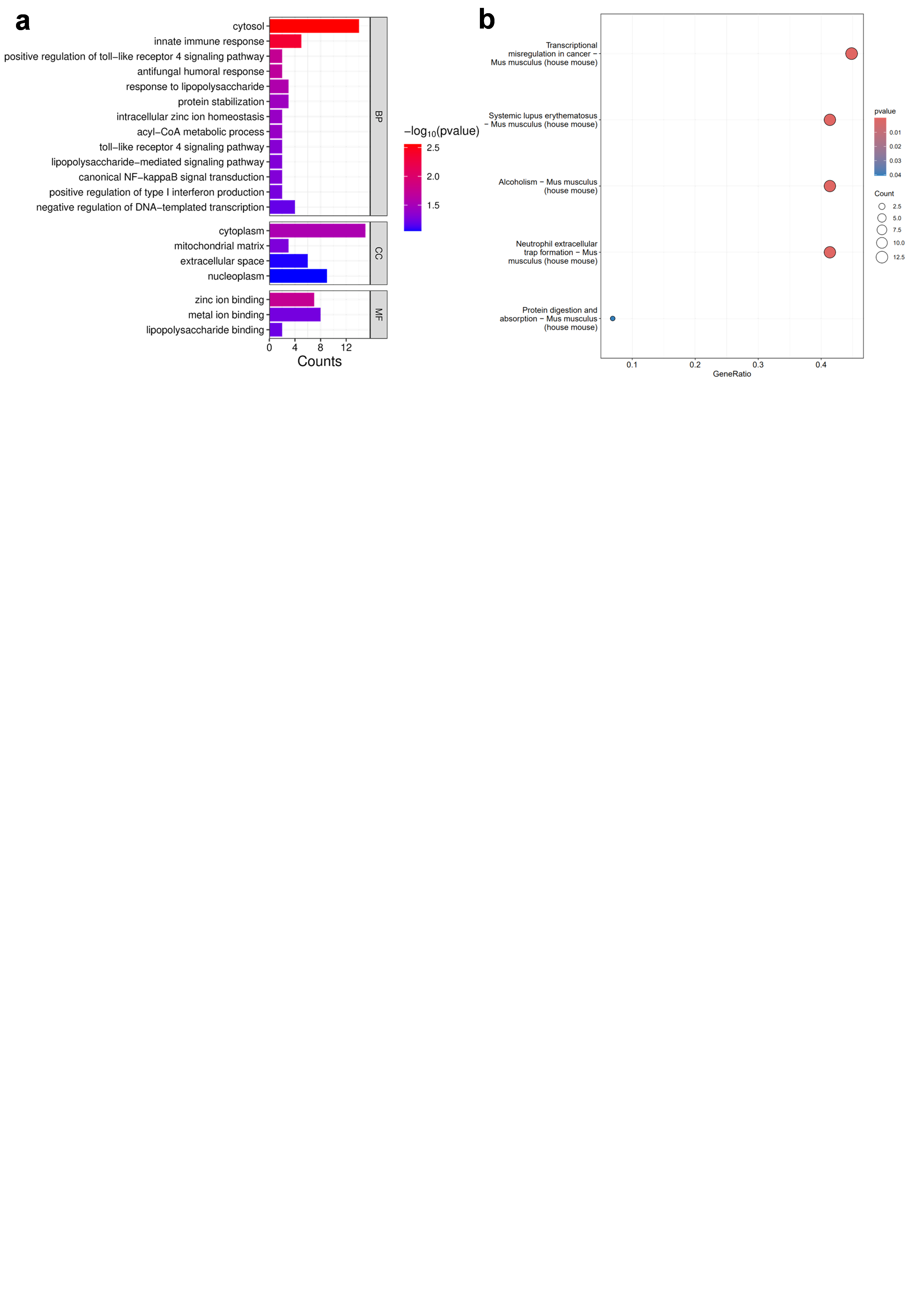

Supplement: Supplementary file 1 [file biomedicines-13-02183-s001.zip › Fig. S2.tif]
